# Improving Suicide Prevention in Dutch Regions by Creating Local Suicide Prevention Action Networks (SUPRANET): A Study Protocol

**DOI:** 10.3390/ijerph14040349

**Published:** 2017-03-28

**Authors:** Renske Gilissen, Derek De Beurs, Jan Mokkenstorm, Saskia Mérelle, Gé Donker, Sanne Terpstra, Carla Derijck, The SUPRANET (Suicide Prevention Action Network) Research Group, Gerdien Franx

**Affiliations:** 1 Department of Research, 113 Suicide Prevention, 1100 CE Amsterdam, The Netherlands; j.mokkenstorm@113.nl (J.M.); s.terpstra@113.nl (S.T.); 2 Netherlands Institute for Health Services Research (NIVEL), 3513 CR Utrecht, The Netherlands; d.debeurs@nivel.nl (D.d.B.); g.donker@nivel.nl (G.D.); 3 Department of Psychiatry, EMGO Institute for Health and Care Research, VU University Medical Center, 1081 BT Amsterdam, The Netherlands; 4 Department of Research & Innovation, GGZ inGeest, 1070 BB Amsterdam, The Netherlands; 5 Public Health Service (GGD) Kennemerland, 2015 CK Haarlem, The Netherlands; smerelle@ggdkennemerland.nl; 6 Department of Implementation, 113 Suicide Prevention, 1100 CE Amsterdam, The Netherlands; c.derijck@113.nl (C.D.); info@113.nl; g.franx@113.nl (G.F.)

**Keywords:** implementation, suicide prevention, multi-level, community approach, European Alliance against Depression

## Abstract

The European Alliance against Depression (EAAD) program is to be introduced in The Netherlands from 2017 onwards. This program to combat suicide consists of interventions on four levels: (1) increasing the awareness of suicide by local media campaigns; (2) training local gatekeepers, such as teachers or police officers; (3) targeting high-risk persons in the community; and (4) training and support of professionals in primary care settings. The implementation starts in seven Dutch pilot regions. Each region is designated as a Suicide Prevention Action NETwork (SUPRANET). This paper describes the SUPRANET program components and the evaluation of its feasibility and impact. The findings will be used to facilitate the national implementation of EAAD in The Netherlands and to add new findings to the existing literature on EAAD.

## 1. Introduction

Suicidal behaviour is a global health problem, with over 800,000 people a year dying by suicide [1]. Suicide and suicidal behaviour are the end result of the complex interaction between social, cultural, biological, and environmental factors [2]. The implementation of a multilevel suicide prevention approach potentially increases the success in reducing suicide and is preferred above single, standalone approaches [3,4]. The World Health Organization (WHO) has deemed suicide prevention a global imperative and recommends the development and implementation of a national strategy, in which health and social sectors collaborate. Within national prevention efforts, local communities play a central role in suicide prevention strategies. They can organize social support, prevention, and continuity of care, especially with regard to high-risk individuals, such as middle-aged unemployed men [5].

In 2004, the European Alliance against Depression (EAAD) was founded. The goal was to create a network of experts from different European countries to implement action-focused, community-based public health interventions aimed at depression treatment and suicide prevention [6].

The shared multilevel approach that is currently being disseminated by the EAAD consortium is based on a successful regional intervention in Germany called the Nuremberg Alliance against Depression (NAAD). NAAD was a two year action program combatting depression and suicide in Nuremberg and consisted of interventions on four levels: (1) increasing the awareness of depression by local media campaigns; (2) training local gatekeepers, such as police officers; (3) targeting high-risk people in the community; and (4) training and support of professionals in primary care settings. NAAD resulted in a sustainable decline in suicidal acts (completed and attempted suicides combined) of 24% compared to baseline [6,7,8]. This community-based, four-level intervention has since been implemented in a range of other European countries such as Hungary, Germany, Portugal, and Ireland via the EAAD consortium. Comparable effects to the NAAD were found in Hungary, for example, where a significant decrease in the suicide rate (56%) was observed in the intervention region, which was significantly greater as compared to the control region [9].

As part of the Dutch National Agenda for Suicide Prevention of the Dutch Ministry of Health, 113 Suicide Prevention, the Dutch suicide prevention centre, is introducing the EAAD model in The Netherlands from 2017 onwards. The implementation starts in seven Dutch pilot regions. Each region is designated as a Suicide Prevention Action NETwork (SUPRANET). This paper describes the SUPRANET program components and the evaluation of the feasibility and impact of SUPRANET.

## 2. Program

### 2.1. Selection of Regions

From January 2016 to August 2016, 113 Suicide Prevention has selected seven pilot regions within The Netherlands to implement the Dutch version of EAAD, called the Suicide Prevention Action NETwork (SUPRANET) Community. A SUPRANET Community is defined as a network of local multidisciplinary teams and organizations with shared ownership and responsibility for preventing suicide within a geographical area. The participating communities are committed to embark on a population-focused approach based on the EAAD model to prevent suicidal acts within their population. Members of the network acknowledge that reducing suicidal acts successfully requires concerted action.

**Figure 1:**
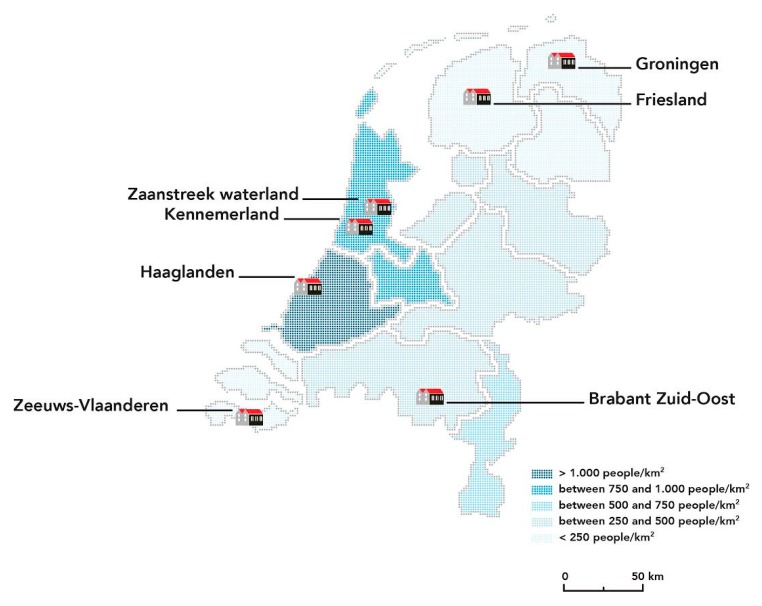
The 7 SUPRANET communities. Colouring represents the population density of the region.

Initially, eight regions participated in the selection. One region decided not to continue the selection process because of the lack of commitment and funding. The SUPRANET communities (Figure 1) were selected based on the following criteria:

- Commitment to suicide prevention expressed by the city council, which is prepared to invest financially;
- Commitment of local stakeholders in and outside health care to be part of the local network and implement the EAAD components and measure progress;
- The potential to create a steering group, local project leader and project teams consisting of at least the following key players:
  ○ General practitioner (GP) practices, including GPs and their mental health support staff (POH)
  ○ social community teams
  ○ mental health care organization
  ○ emergency department
  ○ citizens/consumer/carer representation
  ○ schools and/or employment/social care agencies depending on the high-risk groups targeted by the community.

Furthermore, based on international experiences with the EAAD model, each region had to cover a substantial population, preferably of at least 150,000 inhabitants [10].

### 2.2. Intervention: EAAD Approach

**Figure 2:**
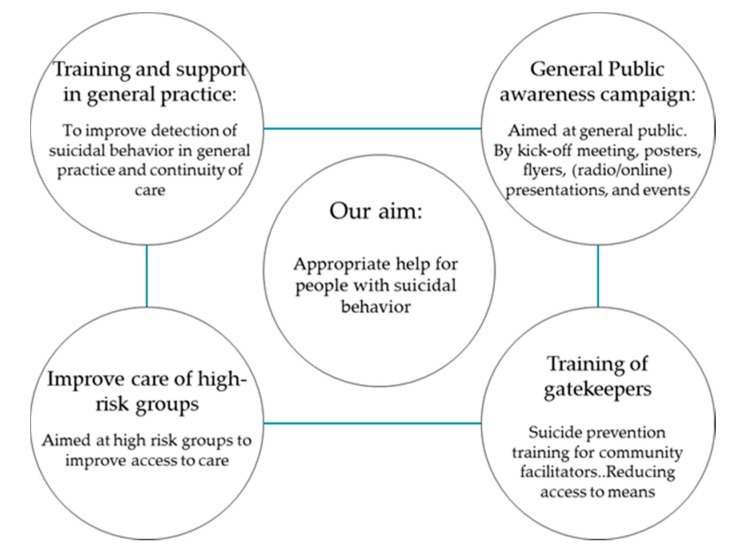
The four level approach of SUPRANET Community, based on the European Alliance against Depression (EAAD).

In January 2017 SUPRANET was launched in seven regions in The Netherlands. The intervention of SUPRANET will consist of the implementation of the EAAD model, a two year multi-component multi-setting suicide prevention package with the four components as shown in Figure 2. A summary of each component is outlined below.

#### 2.2.1. Component 1: The Public Awareness Campaign

The public awareness campaigns in the SUPRANET communities will be based on the national campaign launched by 113 Suicide Prevention from February 2017 onwards. This campaign aims to encourage the general public to talk about suicide and to break the taboo. The core message of the Dutch campaign is “Ask the question of your life”. The campaign has three waves in 2017: the first started in February, the second will start in May and the third in September. Based on this national awareness campaign of 113 Suicide Prevention, the SUPRANET communities will start additional, tailored, regional public awareness campaigns in their own communities in May 2017. This includes the release of more posters, flyers, and (radio/online) presentations, specifically tailored to the community. Regions order the materials at 113 Suicide Prevention, which makes the monitoring of the number of ordered materials feasible. The text on the spreading material is specifically tailored to each community. For example the title is altered, containing the name of the region, or the language or dialect is altered to the most common language or dialect in that region.

#### 2.2.2. Component 2: Gatekeeper Training and Reducing Access to Lethal Means

Gatekeeper training programs are recognized as a successful component in multiple intervention projects [11]. Community gatekeepers are trained to identify suicidal risk, to make contact and “open the gates” for help seeking behaviour [12,13]. The gatekeeper training which is most referred to, is based on the method of Question, Persuade, Refer (QPR). Clinical psychologist Paul Quinnett (CEO QPR institute) created the intervention in 1995. In 2012 the gatekeeper training was translated for Dutch usage [14] and since then many community facilitators, such as teachers, student psychologists, police officers, railway employees, and social workers throughout the country have been trained. Within the SUPRANET communities, 113 Suicide Prevention, and some of the mental health organizations, will provide the training sessions.

This program component also covers collaboration with stakeholders, especially those at suicide hotspots (railroads, high buildings) and implementing interventions to reduce access to means, such as providing professionals information about hot-spots for suicide and (the risks of) medication. This important aspect is based on strong evidence for restricting access to means in prevention of suicide [4].

#### 2.2.3. Component 3: Improve Care of High-Risk Groups

Within each community, specific attention will be dedicated to high-risk groups. Examples of high-risk groups are people who received care in the emergency room after a suicide attempt or middle-aged persons (males and females) between 40 and 65 years of age without paid work who receive social assistance or incapacity benefits. In The Netherlands this latter group has a five- to eight-times higher risk of suicide as compared to persons with paid work [5]. This group can be identified by benefit or unemployment agencies. Apart from these groups, regions may prefer to target an additional high-risk group, such as young persons with vulnerabilities due to lesbian/gay/transgender/bisexual (LGBT)- or ethnicity-related issues. During the project, regions will implement interventions that aim to identify and proactively screen members of high-risk groups, and organize access to care for those in need of help. Regions are encouraged to implement evidence-based interventions, which will be exchanged in “best-practice meetups”, organized by 113 Suicide Prevention. However, regions can also opt for expert opinion-based interventions, for instance, successful interventions developed and tested in different, but similar, programs and contexts.

#### 2.2.4. Component 4: Training and Support in General Practice

SUPRANET has a strong focus on change in recognition and treatment of suicidal behaviour in Dutch primary care. Therefore, crucial to each community, is the participation of general practice, notably the general practitioners (GPs) and their mental health support staff (POH). This is a relatively new professional in most Dutch general practices, most often with a nursing or psychological background, providing brief coaching sessions to patients after being diagnosed by the GP. We aim to recruit at least five GP practices within each region. As part of the intervention, a suicide prevention training package is offered to primary care staff in 2017–2018, stimulating the exploration of suicidal ideation and concrete suicide plans among patients. In addition, the practices will work on continuity of care by improving collaboration with social community teams, emergency rooms, and mental health care.

### 2.3. Tailored Intervention

The four-level intervention will be implemented in each SUPRANET community, specified and tailored to local circumstances and to pre-existing suicide prevention structures or interventions. As such, it can be seen as a combination of a top-down and bottom-up approach. This is in line with the EAAD literature, which states that a participatory approach that maximizes and complements what was already in place, is an incentive for participants to become involved in the process [10].

Tailoring includes the option for each region to fine-tune each of the four SUPRANET components and enrich them with local strategies, based on an analysis of the local situation, thus developing a context-specific suicide prevention plan. An example of such fine-tuning is the selection of gatekeepers with whom one might want to work. These could include teachers, youth workers, and sports coaches in an area where the focus is on suicide prevention among youth. When focusing on middle-aged persons without employment, selected gatekeepers could be professionals within employment agencies, social security agencies, debt, counsellors or clergymen. Examples of tailoring the program component in general practice primary care are: the add-on of a depression prevention e-learning module, or the availability of a case manager to ensure continuity of care after hospitalisation.

## 3. Evaluation of the Feasibility and Impact of SUPRANET

The purpose of the evaluation is to investigate the activities of SUPRANET by examining its feasibility and impact on suicidal ideation and behaviour.

This study aims to answer:

(1) Does the implementation strategy of SUPRANET lead to the expected EAAD outcomes [6,7,8,9] in terms of:
  (a) Reduced suicidal ideation and behaviour and psychological distress?
  (b) Reduced stigma and taboo and improved attitude towards seeking help?
  (c) Increased help seeking behaviour to the helpline of 113 Suicide Prevention?
(2) Is SUPRANET implemented as intended, in terms of:
  (a) What is the reach on each SUPRANET program component (the actors, situations, sites, and contexts involved in the SUPRANET implementation process)?
  (b) What are the barriers and facilitators when implementing the SUPRANET approach?
  (c) What is the impact of the interventions on professional performance?

The evaluation of the feasibility and impact on the Dutch SUPRANET communities will be carried out by the research department of 113 Suicide Prevention, NIVEL (Netherlands Institute for Health Services Research), and local Public Health Services in the participating communities.

### 3.1. Design

The multilevel SUPRANET intervention requires a multilevel evaluation approach. Each SUPRANET program component is part of a multi-layered intervention program, therefore, the effect of one component itself is impossible to discern. Since the primary focus of SUPRANET is on preventing suicide, our primary outcomes studying the impact of SUPRANET are on (attitudes toward) suicidal ideation and behaviour. The outcomes and measurements are described in Table 1. Furthermore, the study on feasibility will consist of a process evaluation; studying the reach of SUPRANET on each program component, a qualitative analysis of the barriers and facilitators when implementing the SUPRANET community approach, and the impact of the program on professional performance.

The outcomes are analysed using a non-randomized controlled pre-post design; i.e., the outcomes are analysed compared to a baseline and to national data not involved in the SUPRANET communities. The process evaluation requires qualitative research with stakeholders in all participating regions.

### 3.2. Evaluation of the Impact of SUPRANET

#### 3.2.1. Suicidal Ideation and Behaviour and Psychological Distress of the General Public

Suicidal ideation and behaviour of the general public will be measured in two ways: First, while challenging, because of the low base rate of completed suicide (11 per 100,000 in The Netherlands), suicide rates will be analysed using data from Statistics Netherlands. Trends in suicide rates during 2017–2019 in the seven SUPRANET communities will be compared to the national suicide trends. In 2015, the number of suicides in the SUPRANET communities was 166, on a population of 1,407,331 (11.8 per 100,000). Differences in suicide rates at baseline and after each implementation year will be calculated using incidence rate ratios. The hypothesis is that the multilevel intervention approach of SUPRANET will lead to a reduction in the suicide rate in the general population, as was found in other EAAD studies [6,7,8,9].

Secondly, the scores on the Suicidal Ideation Attribution Scale [15] in an online survey will be analysed. Four measurements will be carried out using on-line questionnaires among the general population. In each measurement, the national sample will be 1000 and the SUPRANET community sample will be 700 (in each SUPRANET community *n* = 100). The sample will be stratified by gender, age, education, region, and social class. The first measurement, the baseline, took place in January 2017. The second will take place just after the first wave of the public awareness campaign, the third in January 2018, and the fourth in January 2019. A fresh sample is taken for each of the four measurements to prevent learning effects and keep the comparison pure over time. Given an alpha of 0.05 and a power of 0.8, the sample will allow us to detect a small effect size of 0.08. The Suicidal Ideation Attribution Scale (SIDAS) consists of five items that are scored from 0 to 10 and assesses the frequency and controllability of suicidal thoughts, as well as the closeness to a suicide attempt, distress and interference with daily activities. We will investigate, using General Linear Model (GLM) repeated measures, if the changes measured in the SUPRANET communities are more improved than the changes measured nationally.

Furthermore, a secondary analysis will be performed on data of the cross-sectional survey Health Monitor 2016. This survey was carried out by all Public Health Services in The Netherlands between September and December 2016 to provide insight into the physical and mental health and lifestyle risks of adults and senior inhabitants. Participants were randomly sampled from municipality registries. Individuals were excluded if they lived in nursing homes, mental health institutions, or if they were imprisoned or were staying in asylums. Based on a sample calculation which took into account the size of the total target population, as well as the expected response rate, approximately 1.1 million inhabitants of 19 years and older were approached. Mental health was assessed by using the validated Dutch version of the 10-item Kessler Psychological Distress Scale (K10). The K10 uses a five-point Likert scale to measure psychological distress, whereby a higher score indicates more distress [16]. The procedure of the Dutch Health monitor 2016 will be repeated in 2020.

#### 3.2.2. Public Attitudes, Stigma, and Taboo

The online survey among the general public partly replicates the baseline questionnaire for the general public of the Optimising Suicide Prevention Programmes and Their Implementation in Europe (OSPI-Europe) study [19], with Dutch translations of the Depression Stigma Scale [17] and the Attitude Towards Seeking Professional Psychological Help short form [18] (Table 1). We added questions about (talking about) suicidal behaviour, experience, taboo, and the Suicidal Ideation Attribution Scale (SIDAS) [15].

Changes in help-seeking behaviour, stigma, talking about suicide, and taboo around suicide will be analysed using GLM repeated measures. We will compare the results over time, as well as time × effect interactions to reveal if a SUPRANET community is differently (more) effective than the nationally-observed changes.

#### 3.2.3. Help-Seeking Behaviour to the Dutch Suicide Prevention Crisis Service 113

In addition to measuring an intervention effect within the general public with self-reporting, an intervention effect will also be measured by analysing the volume of crisis calls to the helpline of 113 Suicide Prevention. A significant increase in monthly calls during and just after the three waves of the public awareness campaign is hypothesized compared with the same months in the previous year [20]. Furthermore, due to the multicomponent approach, we expect bigger increases in crisis calls in the SUPRANET regions compared with the control region (i.e., national data outside the SUPRANET communities) [20]. This is feasible since the place of residence of each caller is monitored.

### 3.3. Evaluation of the Feasibility and Process of SUPRANET

In addition to evaluating the impact of the SUPRANET intervention in the ways described above, we will conduct an evaluation with the aim to: (1) generate an overview of the actors, situations, sites, and contexts involved in the SUPRANET implementation process and the main implementation activities undertaken within the regions; (2) create an insight into the degree to which suicide prevention is actually embedded into routines, or “normalized” in daily practice and routines, identify the barriers and facilitators to and in implementing the four-level intervention, and illuminate recommendations for broader dissemination of the EAAD approach in The Netherlands; and (3) study the impact of the SUPRANET interventions on professional performance.

#### 3.3.1. Overview of the Reach of the SUPRANET Components, Actions, and Interventions in Each Community

To generate an overview of the reach of SUPRANET, and to reveal intermediate effects on the outcomes, all specific actions and interventions related to the four components of the SUPRANET approach in each of the intervention regions are monitored. For the public awareness campaign, the amount of posters, flyers, etc., for each region is registered, as well as the location. Furthermore, questions on awareness and recognition (seen, read, or heard) the media campaign are asked in the general public in the online survey. Another example of monitoring the reach of each SUPRANET component in each region is the number of community facilitators that are trained as gatekeepers.

Every six months (November 2016, May 2017, November 2017, May 2018, and November 2018) we will ask the regional implementer of each SUPRANET community to fill in an online survey, with questions about the actors, sites, and contexts involved in the SUPRANET implementation process and the main implementation activities undertaken within the regions according to the Re-Aim Model (Reach, Effectiveness, Adoption, Implementation, Maintenance). Since it is possible for each region to fine-tune each of the four SUPRANET components and enrich them with local strategies, thus developing a context specific suicide prevention plan, the survey also monitors additional local strategies and interventions. Additional local interventions will be monitored by 113 and evaluated by the epidemiologists working at the local Public Health authorities.

#### 3.3.2. Identifying the Barriers and Facilitators to, and in Implementing, the Four-Level Intervention

During the process evaluation the Normalization Process Theory (NPT [21]) will guide our decisions about the predominantly qualitative data collection and analysis. NPT is designed to evaluate the effect of multi-component interventions in a dynamic environment. NPT encourages researchers to follow implementation processes, by focusing on a range of people, situations, times, and places that are involved in all aspects of enacting that process. NPT helps to make sense of collective, distributed, patterns of work.

NPT helps to classify complex implementation work that people do around four conceptual constructs: coherence, cognitive participation, collective action, and reflexive monitoring. Coherence refers to how people individually and collectively make sense of the situation when they are faced with the problem of operationalizing complex interventions, in our case suicide prevention activities. It implies that the actors have a shared understanding of what constitutes the suicide prevention work. Cognitive participation is the relational work that people do to build and sustain a community of practice around the suicide prevention work. Collective action is about how suicide prevention is organized and what factors constrain and structure the activities. The fourth mechanism of reflexive monitoring is about the appraisal of the suicide prevention work and the modifications the actors may propose to make the suicide prevention activities workable in practice.

We will purposefully select settings and individuals within the SUPRANET communities to participate in the process evaluation. This means that we will select respondents beyond the “usual” respondents, like the health professional or patient. We will focus on others who are vital within suicide prevention, those that are less visible and often omitted from implementation studies. For example, receptionists in GP practices, debt counsellors, clergymen, and sports coaches. They have to accommodate and realise aspects of adequate suicide prevention. NPT also recommends selecting a large range of sites and contexts, beyond the usual situations, times, and contexts of health care research. We will follow the regional networks for 18 months during their implementation journey in different situations and places: the local project management table, local health authorities or mayor’s offices, GP practices, social community teams, the benefits payment offices, and so forth. We will also pay attention to the diffuse situations where suicide prevention work is discussed, complained about, or praised.

Methods of data collection for this part of the evaluation are as follows:

(1) Every three months semi-structured interviews will be conducted with the seven project managers in charge of the overall coordination within each SUPRANET community, and (at least) three other relevant participating stakeholders (to be selected purposefully at every interview period) per community. The interviews will focus on the implementation processes and activities and the facilitators and barriers of and to the implementation process per program component. Over the course of the intervention, we will interview and survey SUPRANET participants from different backgrounds and settings: those involved in the local implementation/project teams and those involved in the organisations that will actually implement the suicide prevention interventions. The interviews will be analysed using the NPT framework.
(2) During the period of the SUPRANET community program (2017–2018) relevant documents supporting the implementation process of the regions will be analysed (such as work plans, policy documents).

Since, the interviews will be conducted every three months with 3–5 persons per region, surveys will be held every six months with all seven local project leaders, and every six months the project environment will be screened for relevant documents regarding the implementation process; after 18 months this will lead to a total of approximately 125 to 200 interviews, 21 surveys, and a number of documents produced by the regions.

We will use all data sources to perform the analysis and answer our research questions (triangulation). The surveys will be analysed by listing actors and settings, barriers and facilitators, and by thematic analysis of the qualitative information. The interviews will be coded using thematic analysis, and grouped under the various NPT constructs and components. Thematic analysis consists of a process of coding in six phases to create established, meaningful patterns. These phases are: familiarization with data, generating initial codes, searching for themes among codes, reviewing themes, defining and naming themes, and producing a scientific publication and recommendations for broader dissemination of the program in The Netherlands [22].

#### 3.3.3. Impact of the Intervention on Professional Performance: Gatekeepers Attitude, Knowledge, and Skills

The gatekeeper intervention will be evaluated within the SUPRANET communities with measurements before and after the gatekeeper training sessions. We will use a baseline questionnaire prior to the start of the training and a follow-up questionnaire six weeks after. This is already a standard procedure for the gatekeeper training sessions given by 113 Suicide Prevention.

Gatekeepers’ identification of people with suicidal thoughts, referrals to professional care, knowledge of suicidal behaviour, and confidence in their abilities to talk about suicide will be evaluated with an instrument based on other gatekeeper training surveys [23], added by questions assessing confidence on talking about suicide [24]. We hypothesize that the training has a significant impact on gatekeepers’ referral behaviour, knowledge of suicide prevention activities, and confidence in their abilities to ask about suicide [23,24]. Furthermore, we expect that within the SUPRANET communities, relatively higher numbers of gatekeepers will be trained when compared to national numbers.

#### 3.3.4. Impact of the Intervention on Professional Performance: Identification of Depression and Suicide (Attempt) within Primary Care

Using the NIVEL primary care database (NIVEL-PCD) we can extract incidence and prevalence rates of depression and suicide (attempt) for individual general practice. NIVEL-PCD is one of the largest primary care databases in Europe, covering more than 500 GPs with a total practice population of 1.6 million individuals. NIVEL-PCD collects routine electronic health record data, including health problems, consultations, prescriptions, and referrals. Health problems are ICPC coded (international classification of primary care) [25]. The database is representative of the Dutch population. If practices are participating in the intervention, but not yet connected to the NIVEL-PCD, we will include the practices in the NIVEL PCD for the time of the study. Within participating GP practices in all seven regions we will collect all registrations of P76 (depression), P03 (depressed feeling) and P77 (suicide (attempt) and compare the rates 12 months before the start of the project (January–December 2016) to those 12 months after the end of the two year program (January–December 2019). An increase in incidence is hypothesized, due to an increase in recognition of depression and suicidal behaviour within primary care after the suicide prevention training offered to GPs and mental health nurses in the SUPRANET communities. Within the GPs, for the year 2015, an incidence of 11.4 per 1000 patients with P76 (depression) was found, and an incidence of 9.6 for P03 (depressed feeling) For a standard practice with about 2500 patients, we expect around 30 new cases of P76 per year and 25 new cases of P03 per practice. Within one region with five GPs, we expect 275 new registrations of depression and depressive thoughts. Within the seven regions we expect around 1900 new cases of depression. Within the NIVEL registration, we will use a matched control group of around 30–40 practices. Given an alpha of 0.05 and a power of 0.8, this will allow us to detect a small effect size of 0.10.

Analysis will be carried out using multilevel regression techniques, with the incidence of depression and suicidal thoughts as the dependent variable and the condition (intervention or control) as the independent variable. Covariates will be the characteristics of the practices (urban/rural, distribution of age, solo practice, or group practice) and the incidence of depression and suicidal thoughts before the intervention. The different levels are the region, the general practice and the patient.

Furthermore, to measure change in guideline adherence, we will look at the key recommendation of the Dutch guidelines, i.e., the exploration of suicidal ideation and concrete suicide plans by GPs and/or primary care nurses [26,27] within the group of depressed patients. We will measure this by making use of a procedure similar to the one used within the sentinel practices of NIVEL Primary Care Database. Monthly, all new contacts for P76 (depressive disorder) within the electronic medical record of the intervention practices will be extracted by a research or doctor’s assistant. The GP or the mental health nurse will be asked to retrospectively answer an additional short questionnaire for each patient about whether or not suicidal thoughts have been explored, what was the level of intensity of the thoughts and concreteness of suicidal plans, and what actions/interventions were proposed/agreed upon. The questionnaire has been developed by experts and based on the recommendations of the Dutch suicide prevention guideline [26], and are added in supplement 1. These are basic elements of the gatekeeper training offered to GPs and mental health nurses in the SUPRANET communities. We will compare the answers of the intervention practices with data collected within a non-randomized group of 40 sentinel practices, a nationally representative subgroup of practices participating in NIVEL Primary Care database with in total 120,000 registered patients (0.7% of the Dutch population). Via these sentinel practices, NIVEL will collect additional data on suicide and suicide attempts over the last 30 years. In these practices an electronic tool incorporated in the EMD of the GP, the P-module, asks for additional information from the GP triggered by the use of an ICPC code, in order to discriminate between cases and non-cases. If a case is defined, the application generates an additional electronic questionnaire about the characteristics of the patient and the content of the GP encounter. In the past 30 years such an additional questionnaire has been used in suicidal patients after fatal and non-fatal suicide attempts indicated by ICPC-code P77 used by the GPs [28]. For the present study a new category has been defined with an extension of the P-module and an extra electronic questionnaire related to TCPC code P76.

We hypothesize that, within the intervention practices, suicide ideation will be more often explored within depressed patients after the suicide prevention training offered to primary care staff (2017–2018) as part of the intervention, when compared to the period before the training (2016) and when compared to those sentinel practices who are not part of the intervention group and did not have the training.

## 4. Discussion

113 Suicide Prevention has the ambition to implement SUPRANET nationwide. The results of this study will help other regions to more effectively tailor the program to their context, important for the national implementation of the EAAD in The Netherlands. The results will be of high relevance for countries in and outside of Europe.

As suicides have been rising since 2007 in The Netherlands, 113 Suicide Prevention believes it is time to really tackle the problem, and directly target suicidal behaviour. While the focus of the public awareness campaign of the EAAD lies on depression, with the secondary aim to reduce suicidal behaviour, in SUPRANET we decided to directly focus on suicide. We believe the specific focus fits well in the Dutch culture, since the Dutch expect others to be open and direct. Furthermore, general practitioners and gatekeepers in The Netherlands are confronted with suicidal behaviour more often than before. Therefore, they understand the need for an intervention with a specific focus on suicide prevention. Regarding the public awareness campaign, we know from the literature that properly communicating about suicide can actually result in less suicidal behaviour (Papageno effect). 113 Suicide Prevention appoints to the subject suicide direct and encourages people to talk about it. Still, as it is a novum to offer a public campaign on suicide prevention in The Netherlands, a safety procedure is built in. As soon as any rise in adverse events are reported, either via the national railways or via other monitors, such as social media, the campaign will immediately be aborted.

Compared to existing studies on the EAAD, our study has a strong focus on change in recognition and treatment of suicidal behaviour in Dutch primary care. Since GPs play a crucial role in Dutch mental health care and in our suicide prevention intervention, and because the NIVEL primary care database provides good research opportunities in terms of reliable data collection, our project provides a unique chance to assess the effectiveness of the program within primary care. Ideally, we would also be able to collect data from other health care partners, such as social community teams or specialized (mental) health care. However, the results will offer unique insight in the way GPs deal with suicidal ideation and behaviour in depressed patients and will learn how to optimize the training of GPs. Additionally, once the NIVEL depression module has been constructed, it can be used for a longer period of time with only limited funding, allowing us to assess changes in suicide prevention within primary care over years to come.

Regarding the qualitative analysis, previous studies have done quite a large amount of work analysing the success and barrier factors of the EAAD program. Still, no overall list of success and barriers factors has been published. By doing so, our study hopes to facilitate the further implementation of the EAAD across Europe and beyond.

## 5. Conclusions

This paper describes the implementation of the European Alliance against Depression (EAAD) by creating local Suicide Prevention Action Networks (SUPRANET) in seven Dutch pilot regions and how we plan to evaluate it. The evaluation consists of both quantitative and qualitative analyses. The findings will be used (1) to facilitate the national implementation of EAAD in The Netherlands; and (2) to add new findings to the existing literature on EAAD.

## Figures and Tables

**Table 1:** Outcomes and measurements per target group.

Evaluation	Target Group	Compared to Baseline and to National Level, Does Being a SUPRANET Community Have an Impact on:	Measurement	Source
Part 1	General Public	Severity of suicidal ideation and behaviour	Suicide trends	Statistics Netherlands
			Suicidal Ideation Attributes Scale [15]	Online Survey 113
		Psychological distress	Kessler Psychological Distress Scale (K10) [16]	Dutch Health Monitor Public Health Services
		Public attitudes, stigma and taboo	Depression Stigma Scale [17]	Online Survey 113
			Attitude towards seeking professional psychological help short form [18]	Online Survey 113
			Questions on suicide experiences and perceived taboo	Online Survey 113
		Help-seeking behaviour	Change in monthly crisis calls to 113 during intervention compared with previous year	113
Part 2	Community facilitators	Identification of people with suicidal thoughts and linking to professional care	Number of people screened and linked to professional care in previous month	113
		Attitude and confidence	Questions on attitude toward suicide and confidence in assessing and managing suicidal patients	113
	General Practitioners	Identification of patients with depressive symptoms and suicide(attempt) in primary care	Change in recognition of depression and suicidal behaviour within primary care	NIVEL/Primary Care Database Sentinel Practices
		Compliance to professional standards and guidelines regarding the exploration of suicidal behaviour with patients presenting with depressive symptoms	Questions on the exploration of suicidal behaviour and what actions/interventions were proposed/agreed upon. The questions are added in Appendix A.	NIVEL/Primary Care Database Sentinel Practices

## Appendix A. Suicide Prevention within Primary Care-Scale

Q1 You registered a patient with a depressive disorder (ICPC 76). Is this a new episode?
  ❍ Yes
  ❍ No
Q2 Did you ask the patient if he/she had suicidal thoughts?
  ❍ Yes(GO TO Q3)
  ❍ No (GO TO Q16)
Q3 What did the patient answer?
  ❍ Yes, frequently, (WHEN NO NEW EPISODE (Q1 = NO), GO TO Q6)
  ❍ Yes, sometimes (WHEN NO NEW EPISODE (Q1 = NO), GO TO Q6)
  ❍ No ((WHEN NO NEW EPISODE (Q1 = NO), GO TO Q16, WHEN NEW EPISODE GO TO Q4–Q9, AND THEN TO Q16)
Q4 Do you know if the patient has a history of suicidal thoughts or behaviour?
  ❍ The patient has a history of suicidal thoughts or behaviour
  ❍ The patients has NO history of suicidal thoughts or behaviour
  ❍ I do not know that
Q5 Do you know if there is a family history of suicidal behaviour?
  ❍ There is a family history of suicidal behaviour
  ❍ There is NO family history of suicidal behaviour?
  ❍ I do not know that
Q6 Did you ask the patients if he/she lately has been worrying a lot about his/her problems and/or about dying?
  ❍ Yes, the patient has been worrying a lot about his/her problems and/or about dying
  ❍ No the patient has NOT been worrying a lot about his/her problems and/or about dying?
  ❍ I do not know that
Q7 Do you know if the patient experiences hopelessness?
  ❍ Yes, the patient experiences hopelessness
  ❍ No, the patient does not experience hopelessness
  ❍ I do not know that
Q8 Does the patient feel that he/she is a burden to others?
  ❍ Yes, the patient feels he/she is a burden to others
  ❍ No, the patient does NOT feel he/she is a burden to others
  ❍ I do not know that
Q9 Does the patient feel some kind of entrapment?
  ❍ Yes, the patient feels entrapped
  ❍ No, the patient does not feel entrapped
  ❍ I do not know that
Q10 Did you, or do you intend to involve significant others to discuss the patient’s suicidal thoughts?
  ❍ Yes
  ❍ No
Q11 Did you discuss with the patient how he/she can involve significant others to help the patient cope with his suicidal thoughts?
  ❍ Yes
  ❍ No
Q12 Do you know if there are recent stressors (lay-off, divorce) that might be related to the current suicidal thoughts?
  ❍ Yes, there are recent stressors (lay-off, divorce) that might be related to the current suicidal thoughts?
  ❍ No, there are NO recent stressors (lay-off, divorce) that might be related to the current suicidal thoughts?
  ❍ I do not know that
Q13 Do you know if the patient has made concrete plans/preparations (such as collecting medication, or buying a rope) to kill himself?
  ❍ Yes, the patient has made concrete plans/preparations to kill himself?
  ❍ No, the patient has made NO concrete plans/preparations to kill himself?
  ❍ I do not know that
Q14 Do you know if the patient trusts himself?
  ❍ Yes, the patient trusts himself
  ❍ No, the patient does NOT trust himself
  ❍ I do not know that
Q15 Did you feel confident enough to ask for suicidal thoughts?
  ❍ Yes
  ❍ No
Q16 Could you indicate why you did not ask for suicidal thoughts?
  ❍ I thought the patient would not be suicidal
  ❍ There was not enough rapport to ask such a sensitive question
  ❍ The patient indicated earlier that he was not suicidal
  ❍ I did not want to give the patient any ideas
  ❍ Different namely….. ____________________
Q17 Did you register the presence/absence of suicidal ideation?
  ❍ Yes, in the Electronic patient file
  ❍ No
  ❍ Yes, in the..... ____________________
Q18 Did you:
  ❍ Adopt a wait and see policy
  ❍ Make a follow-up appointment
  ❍ Refer the patient to a mental health nurse within primary care
  ❍ Refer the patient to specialized care
  ❍ Consult the crisis services, or a liaison psychiatrist?
  ❍ Prescribed antidepressants:
    (a) No
    (b) Benzodiazepine
    (c) Antidepressants
    (d) A combination of Benzodiazepine and antidepressants
  ❍ Recommended online help tools
  ❍ Made the patient aware of suicide crisis hotline
  ❍ Different, namely ____________________
Q19 When you referred a patient to specialized care, did you check if the patient indeed checked in?
  ❍ Yes
  ❍ No
  ❍ NA
